# Diclofenac Salts, VIII. Effect of the Counterions on the Permeation through Porcine Membrane from Aqueous Saturated Solutions

**DOI:** 10.3390/pharmaceutics4030413

**Published:** 2012-09-12

**Authors:** Adamo Fini, Glenda Bassini, Annamaria Monastero, Cristina Cavallari

**Affiliations:** 1 Department SMETEC, University of Bologna, Via San Donato 15, 40127 Bologna, Italy; Email: chicchipissy@aliceposta.it (G.B.); annamari_m@hotmail.it (A.M.); 2 Department of Pharmaceutical Sciences, University of Bologna, Via Belmeloro 6, 40127 Bologna, Italy; Email: cristina.cavallari@unibo.it

**Keywords:** diclofenac salts, solubility, partition coefficients, permeation, pig ear membrane, permeation coefficient, counterion effect

## Abstract

The following bases: monoethylamine (EtA), diethylamine (DEtA), triethylamine (TEtA), monoethanolamine (MEA), diethanolamine (DEA), triethanolamine (TEA), pyrrolidine (Py), piperidine (Pp), morpholine (M), piperazine (Pz) and their *N*-2-hydroxyethyl (HE) analogs were employed to prepare 14 diclofenac salts. The salts were re-crystallized from water in order to obtain forms that are stable in the presence of water. Vertical Franz-type cells with a diffusional surface area of 9.62 cm^2^ were used to study the permeation of these diclofenac salts from their saturated solutions through an internal pig ear membrane. The receptor compartments of the cells contained 100 mL of phosphate buffer (pH 7.4); a saturated solution (5 mL) of each salt was placed in the donor compartment, thermostated at 37 °C. Aliquots were withdrawn at predetermined time intervals over 8 h and then immediately analyzed by HPLC. Fluxes were determined by plotting the permeated amount, normalized for the membrane surface area *versus* time. Permeation coefficients were obtained dividing the flux values *J* by the concentration of the releasing phase—that is, water solubility of each salt. Experimental results show that fluxes could be measured when diclofenac salts with aliphatic amines are released from a saturated aqueous solution. Different chemical species (acid, anion, ion pairs) contribute to permeation of the anti-inflammatory agent even though ion-pairs could be hypothesized to operate to a greater extent. Permeation coefficients were found higher when the counterion contains a ring; while hydroxy groups alone do not appear to play an important role, the ring could sustain permeation, disrupting the organized domains of the membrane.

## 1. Introduction

Transdermal delivery of drugs offers important advantages over oral administration, especially when drugs, such as non-steroidal anti-inflammatory drugs (NSAIDs), promote adverse effects in the stomach and intestine. For instance, this route is easy and painless, it protects the active compound from gastric enzymes, and it avoids the hepatic first-pass effect. In addition, it is simple to terminate the therapy if adverse or undesired effects occur. The success of a transdermal drug delivery system depends on the ability of the drug to penetrate the skin in sufficient quantities to maintain therapeutic levels, at least locally near the site of administration. The skin, however, is a natural barrier, and only a few drugs can penetrate the skin easily and in sufficient quantities to be effective. In recent years, numerous studies have therefore been conducted in the area of penetration enhancement. Alternatively, *in vitro* permeation studies were carried out, designing suitable transdermal therapeutic systems in order to achieve the desired permeation of the drug across human skin. The external layer of the skin, the stratum corneum, acts as a hydrophobic barrier for the permeation of most drugs, especially towards hydrophilic or ionized ones. As a consequence, a large percentage of drugs, which are weak acids or bases, are usually used in their un-ionized form to enhance permeability by topical forms. 

Absorption of therapeutic agents involves their penetration into the membrane surface either for local or systemic disease: to achieve the required degree of penetration, not only are the properties of the membranes involved, but also the chemical nature, size and structure, and lipid/water partition coefficient. However, it has also been reported that many acidic non-steroidal anti-inflammatory drugs can permeate in the ionized form, especially under the influence of an electric field [1] and without any external help. The literature reports the permeation of many drugs in the ionized form, as a function of pH or as salts (e.g., salicylates) [2]. It appears, therefore, that the pH-partition hypothesis for the absorption is not exactly followed and that different mechanisms can operate or different pathways for absorption can be important, other than crossing of the lipid regions of the skin. These facts support the possibility of using both forms of an ionizable drug for transdermal delivery. Diclofenac, a potent acidic NSAID of the acetic acid class, has also been widely studied for its transdermal absorption, as an acid and as a sodium salt, from different pharmaceutical forms and in the presence of a variety of permeation enhancers [3,4,5]. To overcome solubility problems, it has recently been introduced into the pharmaceutical market as a salt with aliphatic amines in the form of an emulgel (DEtA salt) or patch (HEPy salt) for topical anti-inflammatory therapy. The ability of these salts to partition towards a lipid phase, such as *n*-octanol, as ion pairs, was reported [6], and that the DEtA salt can be released from a saturated aqueous solution [7] or from different formulations [8,9,10,11]. A range of different aliphatic amines were then employed to prepare new diclofenac salts that offered interesting properties in the solid state (hydration, polymorphism, amorphization) and in solution form [12,13,14,15]. 

Since there is little information available about the effect of the counterions on the permeation of diclofenac salts, some of these salts were employed in this paper to test their ability to permeate a porcine membrane and to evaluate the influence of the counterions on the permeation. We choose diclofenac salts with the following aliphatic amines: mono- (EtA), di- (DEtA) and triethyl- (TEtA) amine; mono- (MEA), di- (DEA), and tri- (TEA) ethanolamine; pyrrolidine (Py), piperidine (Pp), morpholine (M), piperazine (Pz) and the corresponding *N*-(2-ethanol) derivatives (HEPy, HEPp, HEM, HEPz, respectively). In this way, we were able to explore a wide range of structurally related counterions.

Porcine ear skin was used in the present work as an *in vitro* model membrane [16] that simulates penetration through human skin, since a similar penetration for topically applied substances, especially hydrophilic compounds, has also been reported [17,18].

## 2. Results and Discussion

Acidic diclofenac displays very low water solubility in its un-ionized form—this is, related to its high melting point and its intrinsic hydrophobicity—since the only hydrophilic functional group, *i.e*., the carboxyl group, is involved in the formation of a dimer and therefore not readily available to interact with the solvent. Salt forms are often preferred in commercial formulations to obviate solubility problems, even though in the ionized or salt form the permeability of the drug could generally be greatly reduced, due to its lower partition coefficient [19]. It could be concluded that in the case of ionizable drugs, the chemical form of the most suitable candidate for optimum permeation is the un-ionized one, since it has a lower polarity and high logP, which means a higher affinity for the horny layer that has to be crossed for absorption after topical application. This statement has been frequently contradicted. In particular, in the case of diclofenac, salts with aliphatic amines can be partitioned towards an *n*-octanol phase [6], or penetrate through human skin [9,20], even in iontophoretic experiments [1], or in combination with chemical enhancers [4], or by means of special formulations [21,22,23]. Few studies have been performed on the role of the counterions on the permeation/absorption of some diclofenac salts [7,24].

The main purpose of this paper was to evaluate the ability of diclofenac to permeate when in the chemical form of a salt, without any manipulation of the use of the acidic form in the presence of a buffer, or of the sodium salt in the presence of permeation enhancers, or particular formulations different from a simple aqueous solution. The availability of a range of diclofenac salts with aliphatic amines [15] also suggested a study on the possible difference between the sodium and organic cations and possible differences among the counterions present in the salts in driving the permeation. In order to focus the attention on this aspect, it was therefore decided to carry out the permeation tests as simply as possible, avoiding buffered systems in the releasing phase. For the same reason, we used the skin of only one donor in these experiments, and a simplified treatment of the membrane was used. The difference in concentration of each salt in the two compartments was supposed to be sufficient to promote permeation and no enhancer was employed. Finally, since the amount that permeated the skin in preliminary experiments was found to be low, pH control of the solubility was not important in the donor compartment, since only the receiving phase was buffered. 

Transdermal delivery of therapeutic agents involves the penetration of the drug across the external dermal surfaces towards deeper lying tissues. According to this simple scheme, we examined a number of parameters associated with the permeation of diclofenac salts, a knowledge of which is important to interpret the experimental results, such as the chemical nature, solubility and *n*-octanol/water partition coefficient of the salts, and the properties of the membranes involved.

### 2.1. Nature of the Salts

The permeant drug is represented in this paper by a number of diclofenac salts formed with organic aliphatic amines. Diclofenac is a weak acid, poorly soluble in water in its un-ionized form, and mainly formulated as a salt. Commercial salt forms of this drug contain either inorganic (Na and K) or organic (DEtA, HEPy) counterions. Diclofenac salts with aliphatic amines were further studied in an attempt to improve solubility, and their complex behavior is described both in the solid state and in aqueous solution. In this paper, we examined a group of salts where the starting amines carry structurally related (ethyl *vs*. hydroxyethyl; cycle *vs*. *N*-hydroxyethyl cycle) substituents. 

The effect of the counterions in these pharmaceutical salts used for permeation studies starts from the choice of the salt forming agent. The preparation of the salts is quite simple and many of them crystallize as hydrates of various stoichiometry; this is quite important, in view of their stability and their use in an aqueous medium. The salts with HEM, HEPp, HEPz, HEPy display simple thermograms and this fact allows better examination of the identity and purity of these salts using differential scanning calorimetry [15]. Contrariwise, the salts formed with cyclic bases lacking the *N*-substituent show thermograms with melting endotherms associated with a loss of weight, in some cases well before the melting, suggesting complex thermal events that occur at increasing temperature, associated to thermal dissociation of the salt component (the acidic diclofenac and the free amine). Moreover, the diclofenac salts with amines carrying hydroxyethyl substituent in the ring demonstrated an ease of handling with respect to those lacking these groups, without formation of solvate/hydrates or polymorphs. This aspect can simplify the preparation and, in particular, the reliability of the chemical and physical properties, regardless of the crystallization solvent, and could represent a parameter for a preliminary selection of the optimum salt for the permeation tests. For instance, the diclofenac salt with DEtA, marketed in the form of an emulgel, forms a monohydrate. The one with HEPy, present in a commercial plaster for topical anti-inflammatory therapy exists in two polymorph forms. The salt with Pz differs in its composition when prepared in water or in aprotic solvents, such as THF, since it could be a 1:2 or 1:1 salt. The number of water molecules of crystallization in the hydrates range from 1 for MEA, DEtA, Py salts to 2 for HEPy. Polymorphs were found for MEA, Py, DEA and HEPy salts. As a consequence, attention must be paid to these parameters in order to select the chemical forms that are stable in the present conditions—that is, aqueous solutions at 37 °C, suitable for transdermal permeation. Table 1 lists some physicochemical parameters of these salts.

**Table 1:** Permeation parameters of the diclofenac salts.

Diclofenac Salts	*J* (μg cm^−2^ h^−1^)	Q8 (μg cm^−2^) × 10^3^	S_1_ (μg cm^−3^) × 10^3^	*D* (cm h^−1^) × 10^3^	logP (salt)	logP (free base)	S_2_ (mM)	pKa (base)	pH * (sat. sol.)
MEtA	12	9.3	6.1	2	0.01	−0.13	17.9	10.84	7.62
MEA	6.9	5.6	9.9	0.7	0.08	−1.31	26.4	9.50	6.95
DEtA	48	1.9	13.7	3.7	0.17	0.58	35.4	10.75	7.58
DEA	51	1.8	18.0	2.8	0.08	−1.43	44.9	8.97	6.68
TEtA	23	0.6	6.7	3.4	0.85	1.45	16.1	10.75	7.58
TEA	20	0.6	3.4	3.0	0.64	−1.59	7.6	7.76	6.08
M	26	1.1	6.9	3.8	0.35	−0.72	18.0	8.50	6.45
HEM	21	0.3	4.4	4.8	1.04	−0.489 *	9.7	6.89	5.64
Pz	18	0.1	0.4	45	0.67	−1.17	0.59	4.19	4.29
HEPz	160	0.4	12.5	13	0.24	−0.957 *	29.3	9.05	6.72
PP	87	0.6	4.3	20	0.97	0.80	11.3	11.12	7.75
HEPp	82	0.9	10.7	7.7	0.29	0.449 *	25.2	9.66	7.03
Py	42	0.3	2.0	21	0.21	0.46	5.1	11.32	7.85
HEPy	194	1.5	20.2	9.6	0.17	0.076 *	45.2	9.72	7.06
Na				0.58 **					
				1.3 ***					
Acid				3500 ***					

* calculated values; ** ref. [25]; *** ref. [19]; Abbreviations: *J*, flux; Q8, amount permeated after 8h; S_1_ and S_2_, solubility reported in terms of micrograms per milliliter and millimoles per liter.; *D*, permeation coefficient; logP, decimal logarithm of the partition coefficient for the free base or for the diclofenac salt; pKa, negative logarithm of the acidity constant of the base; pH, calculated value of the saturated solution.

### 2.2. Solubility of the Salts

The transmembrane diffusion process is passive in nature and depends on a concentration gradient as the driving force. In the present paper, the saturated aqueous solution of each diclofenac salt was designed as a suitable donor phase for permeation experiments and the solubility of each salt represents the concentration of the releasing phase. 

Solubility values of these diclofenac salts in water at 25 °C are, in most cases, not higher than that of the sodium salt (30 mM), despite, in most cases, the presence of hydroxy groups in the cations, which should increase the entire hydrophilicity of the salts [15]. In fact, the presence of hydroxy groups in the cation is expected to favor solubility in water for salts having structurally related cations, even though the salt with TEA has a lower solubility than the salt formed with the corresponding base lacking in hydroxy groups. Crystal structures, elucidated for some of these salts (see ref. [15] for bibliography), could offer an explanation for this apparently odd aspect: the presence of hydroxy groups largely promotes anion/cation hydrogen bridges in the solid state, compacting the crystal structure and increasing the melting point and therefore decreasing solubility in water. The salts with a hydroxy cyclic cation also display higher solubility. 

The saturated solution, selected to carry out the permeation tests, can ensure the highest thermodynamic activity and the highest driving force for permeation, in view of possible poor permeation ability of ionic compounds. Since saturated solutions of these salts are relatively diluted (see below), their density and viscosity are lower than that of different formulations, such as gels or microemulsions. As a consequence, no parameter other than the simple nature of the salts can affect their permeation [25]. Even for practical purposes, the choice of a soluble salt prevents possible problems in the preparation of formulations destined to topical applications. The following examples outline two opposite situations where solubility represents a problem. The low solubility value for Pz salt is related the formation of a double salt [15], which does not occur in the case of the HEPz salt. However, even the two cations offer a ring of high hydrophilicity and polarity. The high solubility value for the HEPy salt has been extensively discussed in previous papers [26,27], and can originate supersaturated solutions that need a few days to reach the equilibrium value suitable to obtain reliable values for the permeation test. 

Solubility values are reported in terms of micrograms per milliliter in Table 1, employed for *J* and *D* calculation; values in terms of moles per liter are also shown.

### 2.3. Partition Coefficient of the Salts

To provide an estimate of the degree of drug absorption and distribution within the body (lipid phase/aqueous phase), the partition coefficient (in terms of logP) of the permeant molecules is usually measured. *n*-octanol and water or a buffered aqueous solution are considered to be the *in vitro* standard systems to determine the drug partition coefficient between skin and *in vitro* study fluid [28]. LogP measures the relative affinity of a drug between a hydrophobic and hydrophilic vehicle and, when the drug is in the form of a salt, a dominant hydrophilicity of the drug is expected. For instance, the logP of acidic diclofenac is largely positive, *i.e*., the molecule is highly hydrophobic: at 0% ionization it displays a partition parameter about 3000 times higher than that of the ionized (or salt) form, and this ratio was found to be the same when the values of permeability rate constant were considered for the two forms (un-ionized and ionized) of diclofenac [19]. However, it can also be expected that a certain hydrophobicity is retained, even in the ionized form, when the starting un-ionized form is largely hydrophobic: positive logP values were measured for a series of acidic NSAIDs in phosphate buffer pH 6.6—that is, the ionized form [19]—and a logP 0.70 [29] or 0.92 [30] was found for diclofenac sodium. Moreover, for a pharmaceutical salt, these properties can be modified by changing the counterion used to produce the salt. For instance, in the case of ibuprofen salts, a positive logP of the salt with organic bases was found that promotes the rate of absorption (flux) across a membrane. The logP values reported for these salts revealed an interesting behavior, since, despite the ionic character of the starting compounds that originate charged forms in aqueous solution, the logP values measured are positive, demonstrating a dominant hydrophobicity, sufficiently high to ensure a flux across a membrane. This fact suggests that hydrophilicity of the ionic forms is attenuated or fully compensated when both ions are organic and the formation of ion pairs could be hypothesized [30]. Similar results were also found in the case of diclofenac salts. The formation of ion-pairs with the diclofenac anion operates better with organic than inorganic cations and can be appreciated in the presence of a lipid phase that continuously extracts the ion-pair [6]. The experimentally determined logP for the DEtA diclofenac salt was reported to be 0.853 and indicates that the drug possesses sufficient lipophilicity, suitable for its formulation into a transdermal patch, since “the biphasic nature of the drug mimics the biphasic nature of skin, thus ensuring easy penetration through the skin” [9]. The ion-pair formation of diclofenac anions in the presence of bulky alkylamines as model cationic ions was found to increase the solubility in a non-aqueous vehicle, caused by reducing or neutralizing the ionic charge, as well as enhancing the skin permeability [2].

The diclofenac salts used in the present work were prepared with a variety of aliphatic amines that display positive and negative values of their logP (in their deprotonated form), depending on their structure (Table 1). When comparing pairs of linear basis, a negative logP can be easily observed in the base with hydroxy groups indicating high hydrophilicity. Contrariwise, for the pairs Pp/HEPp and Py/HEPy, logP is always positive, while it is negative for M/HEM and Pz/HEPz. No data are available concerning logP values for the cationic form of these amines, but a similar situation to the one discussed above for the anions could be hypothesized, where, despite the ionic character, logP need not necessarily be positive. Therefore, when the anion/cation association was considered, such as that in the salt, the final logP can be positive or negative, depending on the nature of the association. In the case of the salts with the cyclic bases carrying the hydroxyethyl group, it was found that the group is involved in the formation of ion pairs in the solid state of these salts. The presence of this interaction, also in solution, introduces an unexpected variable affecting the logP of the salts and promoting partition. 

A positive logP is expected to promote drug permeation, especially in topical formulation. Due to the ionic structure, a negative logP is, on the contrary, expected for a drug in the chemical form of a salt that could make it unsuitable for permeation. However, in some cases, hydrophilicity of an ionic salt can be compensated or overcome by the possible hydrophobicity of the cation and of the anion, especially when both of them are organic. This was found to be the case with most diclofenac salts with aliphatic amines (Table 1), that therefore can be proposed as forms suitable for permeation tests, particularly those with a ring-carrying cation, having positive logP.

### 2.4. The Choice of Animal Membrane

For the study of *in vitro* permeation of drugs, excised animal skin is frequently used as a replacement for human skin. A recent study [31] provided a side-by-side comparison of various skin types that could serve as a replacement for human skin in *in vitro* penetration studies. It was concluded that pig skin was the most suitable model of those available in the absence of human tissue. The difference in penetration between human and pig skin, depending on the compound, made it possible to appreciate unlikely overestimation of drug penetration into human skin by extrapolation from experiments with porcine skin. Porcine skin was also preferred for permeation of this group of diclofenac salts; the porcine tissue shows similarities and also dissimilarities in skin structure compared to human skin. However, a similar penetration for topically applied substances, especially hydrophilic compounds, was observed [18]. 

A general problem in using frozen-thawed animal membrane is that the hydrophobic nature of the membrane was found to have decreased after these processes, and this fact should increase permeability to polar rather non-polar substances [25], possibly leveling differences or partially obscuring particular structural details of the permeants or of the formulation. In a transdermal drug delivery of a sodium diclofenac study comparing microemulsion and aqueous systems, the influence of membrane storage conditions on the *in vitro* permeability of the drug was reported. It was hypothesized that after the freezing and thawing procedures, the lipid pathways in the skin do not practically change while the aqueous pathways alone in the lipid bilayers are modified and become more diffusible to water influx. This way, the authors explained the more rapid penetration of the drug from aqueous solution rather than from lipophilic vehicles [25].

### 2.5. Permeation of the Salts

Flux values could be measured for permeation of diclofenac sodium: this fact represents an interesting result and indicates that, despite the higher polarity of a salt form, permeation occurs even in the presence of ionized forms. In some cases, the starting form of the drug was the un-ionized one, but associated with a buffer ensuring complete ionization, and thus a system like this is very close to one starting with an aqueous solution of the sodium salt [32,33,34]. These facts supported the idea that permeation could occur without any problem, even with the present group of diclofenac salts. The parameters discussed above appear even more favorable in promoting their permeation across the porcine membrane, and permeation of diclofenac was observed starting from their aqueous saturated solution. In all cases, interesting flux values with the amount permeated after 8 h (Q8) that decreases, passing from salts with linear to those with cyclic cations.

The concentration gradient across the membrane will not initially be linear, as the permeant equilibrates within the tissue. After a sufficient lag-time (about 2 h for all the salts), a steady state will be achieved and the effective permeant concentration will remain constant at all points in the tissue; a linear flux could be measured in each case in the period of 8 h and, in some case, also of 24 h. The flux *J* of diclofenac apparently varies depending on the nature of the starting salt; however, the *J* values for the different salts cannot be compared, since permeation is driven by different starting concentrations of the saturated solution. 

**Figure 1:**
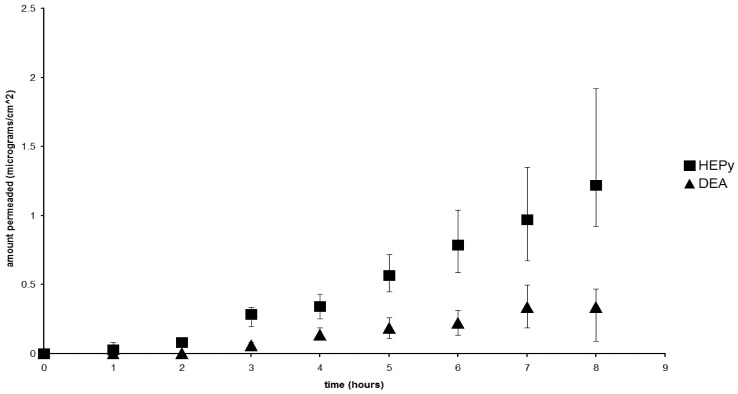
Flux profiles for the diclofenac salts with HEPy (■) and DEA (▲).

**Figure 2:**
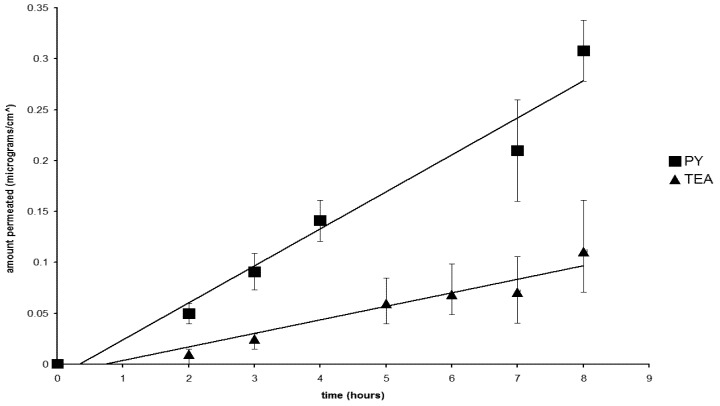
Flux profiles for the diclofenac salts with Py (■) and TEA (▲).

Figure 1 and Figure 2 compare the flux for two pairs of salts, whose solubility, and therefore the concentration of the releasing phase, was found to be very close together. Figure 1 shows the comparison between the HEPy and DEA diclofenac salts, having comparable and high solubility; while Figure 2 offers the comparison between the Py and TEA salts, having lower and similar solubility. In both cases, notable differences could be observed concerning flux values that were found independent of the concentration of the releasing phase and, in the case of the pair Py and DEA salts, higher permeation flux is achieved with the solution containing lower concentration of the salt. This fact can be related to the influence of the counterions. 

Therefore, unless using a releasing phase with equal concentrations for all the salts, the best parameter to consider the effect of the counterions in the permeation process is *D*, the permeability coefficient, which is the flux normalized by the concentration in the releasing compartment that is the solubility of the salts [35].

### 2.6. The Nature of the Permeant Species

A point of discussion could also be the nature of the permeant species. In a solution containing a diclofenac salt with an amine (A), three species can be present containing the anti-inflammatory moiety: the diclofenac anion (D^−^), originated by the dissociation of the salt; the un-ionized acidic diclofenac (DH), originated by the hydrolysis of the anion D^−^: D^−^ + H_2_O ↔ DH + OH^−^; and the ion-pair (D^−^A^+^), originated by the association of anion and cation. The concentration of D^−^ is formally equal to the concentration of the salt, which is relatively high, the releasing phase being a saturated solution, even though, due to hydrolysis and ion association, it could be a little lower than the nominal one. The concentrations of both the other species are difficult to measure but can be calculated and are very low. The concentration of DH is limited by the fact that, in the solution of a salt formed both by a weak acid and base, the pH is independent of the concentration and can be calculated as the mean between the pKa of the two species. In the present cases, the pH of the starting solution can be calculated as ranging from 5.64 for the HEM salt and 7.85 for the Py one and from these values it can be calculated that the concentration of the un-ionized diclofenac existing in these conditions is rather low with respect to the anion. The concentration of ion-pairs is similarly low, being aqueous and of high polarity in the medium where dissociation overcomes association. As a consequence, this rank order of concentration can be expected: [D^−^] >> [D^−^A^+^] ≈ [DH]. However, the hydrophobicity of these species is reversed: DH ≈ D^−^A^+^ >> D^−^, and therefore their ability to permeate the lipid membrane [19]. Acidic diclofenac DH, in fact, is the most hydrophobic species with a logP of 4.4 and a pKa of 4.18 [36]: the low concentration can be more than compensated by the high logP of this species that therefore can be competitive with other permeating species present in the releasing solution [19]. The ability of D^−^ to permeate was reported in the literature [7,19,25] in studies using diclofenac sodium (for which acidic forms or ion-pair formation appear very limited). The skin transport of diclofenac sodium was also achieved in the presence of non-completely aqueous solution and in the presence of high concentration of terpenes as permeation enhancers [4] or from microemulsion [25] or emulgel [37] or other formulations [5] as starting phases or under the application of an electric field [1]. Most of the papers dealing with the permeation of diclofenac salts different from the sodium one suggest that it could occur via formation of ion-pairs [2,6,7,30] formed by undissociated ions of reduced polarity and displaying affinity for a lipid phase (e.g., membrane). For this species too, the ability of the permeation is therefore widely documented. 

As a conclusion, it appears that low concentration of a species is accompanied by a high permeation ability and it is therefore difficult to assess the contribution of each species to whole permeation starting from a diclofenac salt: continuous extraction of the membrane can also further obviate the low concentration of each permeant species. From the present results, it can be confirmed that permeation of diclofenac through a membrane occurs, even when starting from a salt with simple aliphatic amines, and that the chemical nature of the counterion in the salt, as a permeation enhancer, operates with different mechanisms, such as promoting ion-pair formation and modulating its hydrophobicity, affecting the concentration of the more hydrophobic acidic diclofenac and increasing the concentration of the diclofenac anion, by increasing the solubility of the salt.

### 2.7. The Effect of the Counterions

Membrane permeation of pharmaceutical salts occurs when the ionized drug is associated with suitable counterions, which for acidic drugs could be large alkylamines or quaternary ammonium bases, promoters of the formation of a more lipophilic ion-pair. In this form, the salt can achieve the necessary hydrophobicity to drive permeation across a lipid membrane. The present results confirm that important flux values for diclofenac can also be obtained in the form of salts with simpler amines. There are numerous examples of improved bioavailability of poorly soluble drugs when formulated as a salt with amines, like those of the present paper, such as MEA, DEA and TEA with piroxicam [38], MEA with meloxicam [39] or ibuprofen with MEA, DEtA and TEtA [30]. Some studies also reported the ability of diclofenac to permeate when in the form of a salt with HEPP and MEA [36], 2008 or DEtA and HEPy [7,8,9]: however, with a few exceptions, these papers concern permeation of diclofenac salts starting from different formulations than a simple aqueous solution, which could alter the release and often limits the permeation.

This paper reports the effect of a large variety of aliphatic amines used as counterions in the case of diclofenac salts. For comparison, Table 1 also shows the *D* values found in the literature for the sodium diclofenac and the acidic diclofenac [19]. The *D* value measured at 0% ionization (for the acidic diclofenac), is approximately three orders of magnitude higher than that obtained at 100% ionization—that is for the ionized forms of the sodium salt. The *D* value for the sodium salt agrees with those found in other papers for the same form [7,25] and with those found for the present series of diclofenac salts, confirming the ability of salt forms to permeate. Examination of the *D* values (Table 1) make it possible to outline no apparent relevance concerning the presence of hydroxy groups in the cations, with respect to ones lacking these groups. This appears not to agree with what was reported on the role of alcohols in promoting permeation of drugs [40,41]; counterions can actually be considered derivatives of ethanol and therefore as permeation enhancers at high concentrations, especially with highly soluble salts. This aspect could suggest that hydroxy groups are not available for this role but are involved in anion/cation hydrogen bond, promoting the formation of ion-pairs as the main permeant species in these conditions. A possible role could also be played by the membrane that during its preparation could have partially lost the ability to discriminate between polar and non-polar substances [25]. 

Differences in logP of the salts appear very limited, but comparing structurally related pairs of salts, it can be observed that a higher permeation coefficient corresponds to a higher logP of the salt, suggesting that a common mechanism associated with permeation is the hydrophobicity of the permeant species. This fact agrees with the higher effect played by the presence of a ring lacking the hydroxy group in the cation as the main factor responsible for permeation, and could explain why both morpholine-containing cations promote permeation to a limited extent. It was reported that from the examination of the diclofenac salt with HEM, HEPp and HEPz in their solid state [42], the substitution of the methylene group with O or NH in the ring modifies the charge delocalization on the cations (strongly for O and moderately for NH) and this causes differences in solubility among the salts as a result of a better accommodation of HEM in the aqueous phase [42]. In other words, the diclofenac salts with M and HEM display unexpected affinity for the releasing aqueous phase that slows down permeation with respect to the other structurally related salts.

Comparing the permeation coefficient (*D*) values for these salts (Table 1), it emerges that it is probably the presence of the ring in the cation that is the determinant for a relevant permeation coefficient of diclofenac salts with aliphatic amines, while other structural aspects concur only to a minor extent and with a secondary role; it also represents the structural key to promote the formation of larger ion pairs of improved hydrophobicity and possibly a disrupting ability of the organized domains of the membrane that sustain permeation. This aspect was already reported concerning the effect of the *n*-alkyl group of a series of enhancers that were hypothesized to intercalate/partition into the relatively highly ordered region of the membrane lipid bilayer, inducing disorder and increasing fluidity in this region [43]. From this point of view, diclofenac salts with Py, Pp and Pz should be more suitable for transdermal formulations in an aqueous vehicle, thus avoiding non- or partially aqueous media, permeation enhancers or external devices to achieve efficient levels of absorption of the anti-inflammatory agent.

Finally, the present discussion cannot ignore the potential toxicity of the aliphatic amines when used as salt forming agents for pharmaceutical salts, since their cation accompanies the active moiety during the absorption of the ion pairs and their possible side effects cannot be neglected. As a consequence, the above reported results should represent only a basis to further highlight the toxicology of these amines before proposing these diclofenac salts for practical applications. However, results obtained with DEtA and HEPy diclofenac salts [9,33], which are successfully present in pharmaceutical formulations marketed worldwide, should envisage a positive development for other salts of this series.

## 3. Experimental Section

### 3.1. Materials

Acidic diclofenac was a gift (IBSA, Lugano, Switzerland) of pharmaceutical grade. The following bases: monoethylamine (MEtA), diethylamine (DEtA), triethylamine (TEtA), monoethanolamine (MEA), diethanolamine (DEA), triethanolamine (TEA), pyrrolidine (Py), *N*-(2-hydroxyethyl) pyrrolidine (HEPy), piperidine (Pp), *N*-(2-hydroxyethyl) piperidine (HEPp), morpholine (M), *N*-(2-hydroxyethyl) morpholine (HEM), piperazine (Pz), *N*-(2-hydroxyethyl) piperazine (HEPz), were commercial samples of the highest purity grade available (Sigma–Aldrich, Milano, Italy).

### 3.2. Preparation of the Diclofenac Salts

One gram acidic diclofenac was suspended in a small volume of distilled water: the mixture was heated under magnetic stirring. The selected base, dissolved in a small volume of water, was added dropwise to the suspension. After the addition of the base, diclofenac acid dissolved, the pH was checked and, if necessary, a small excess of acid diclofenac was added to neutralize the possible base excess. The final mixture was heated to boiling point, then filtered and allowed to crystallize. The salt was filtered, allowed to dry in air and analyzed by DSC, TGA and Karl Fisher titration to define the possible formation of hydrate and the salt stoichiometry [15] (Table 1).

### 3.3. Solubility Studies

Saturated solubility of diclofenac salts in water was evaluated. Saturated solutions were prepared by adding excess salt to 10 mL distilled water and stirring for 48 h at 25 °C (preliminary studies showed that 48 h is enough to reach equilibrium solubility). After this period, the suspensions were filtered through a 0.45 μm cellulose acetate filter, suitably diluted and analyzed by HPLC. Three determinations were carried out for each sample to calculate the solubility of diclofenac salts. Table 1 displays the solubility values in terms of millimoles per liter; values in terms of micrograms per liter were calculated using the appropriate MW value that take into account the nature of the solid species present in the saturated solution [15].

### 3.4. Determination of *n*-Octanol-Distilled Water Partition Coefficient of the Salts

The partition coefficient study was performed using *n*-octanol as the oil phase and pure water as the aqueous phase. The two phases were mutually saturated on a mechanical shaker at room temperature for 24 h and used for further measurements. A solution of each salt (about 10^−4^ M) was prepared as the aqueous phase. Five milliliter of this solution were then transferred to 10 mL assay tubes containing 5 mL of *n*-octanol. The tubes were stoppered and shaken for 24 h at room temperature to achieve complete partitioning. After careful separation of the two phases, first using centrifugation and then a separating funnel, the concentration of the drug in the water phase was evaluated by means of HPLC. The related value in *n*-octanol was calculated from the difference between the value of the starting aqueous solution and the value after partitioning with the *n*-octanol phase. The partition coefficient of drug *P*o/*w* was calculated as the ratio between these two concentrations: *P*o/*w* = Concentration in *n*-octanol/Concentration in the aqueous phase. Three replicates were used for the concentrations of *n*-octanol-distilled water solutions for partition coefficient calculations (Table 1) [9].

### 3.5. Permeation Experiments

After removing adhering subcutaneous fat, portions (4 × 4 cm) of porcine ear were carefully cleaned with distilled water and stored at −20 °C. Before each permeability experiment, tissue specimens were thawed at room temperature in phosphate-buffered saline (PBS, pH 7.4). Franz-type diffusion cells were used throughout the study and the diffusion barrier (porcine membrane) was mounted between the two chambers and secured with a spring clamp. Thereafter, the specimens were mounted in flow-through diffusion cells (exposed area 9.62 cm^2^) with the stratum corneum facing the donor compartment and the dermis facing the receptor. Before beginning each permeability experiment, tissue disks were equilibrated for 10 min with PBS (pH 7.4) in both the donor and receiver compartments of the diffusion cells. After equilibration, the PBS was removed from the donor compartment and replaced with 5 mL of each diclofenac salt saturated aqueous solution. The solution was covered with a Teflon disk to seal it from the atmosphere. The receptor compartment of the cell was filled with PBS (100 mL). During the experiments, the solution in the receptor phase was maintained at 37 °C by a thermostat and stirred with Teflon-coated magnetic stirring bars. Fifty microliters aliquots were collected from the receptor side at designated time intervals (1, 2, 3, 4, 5, 6, 7 and 8 h; occasionally the permeability test was prolonged up to 24 h). The permeability study was performed under sink conditions, *i.e*., at the completion of each run the concentration of diclofenac in the receiver compartment is negligible compared to that in the donor chamber. The permeated diclofenac salt concentration was on-line determined by HPLC. The cumulative amount permeated per unit area was plotted against time and the linear section of the graph taken as the steady state flux (*J*, μg cm^−2^ h^−1^): lag time was estimated by extrapolation from this line (Table 1). The values reported are the mean of at least five independent experiments.

### 3.6. Permeation Parameters

According to Fick’s second law of diffusion, the total amount of drug (*Qt*) appearing in the receptor solution in time *t* depends on the following parameters: *A*, the effective diffusion area; *C*o, the drug concentration, which remains constant in the donor phase, the releasing compartment being a saturated solution; the diffusion coefficient; *L*, the thickness of the membrane and *K*, the partition coefficient of the drug between membrane and vehicle. At steady state, the flux, *J*, determined from the slope of the steady-state portion of the amount of the drug permeated divided by *A versus* time is given by the equation: *J* = (*C*o*KD*)/*L* = *C*o*D*, where *D* is the permeability coefficient. The lag time values were determined from the *x*-intercept of the linear region at steady state. The permeability coefficient (*D*) was calculated from the steady-state flux *J* and the applied concentration in the donor compartment (*C*o) as follows: *D* = *J*/*C*o.

### 3.7. Analytical Method

The effluent samples, collected from the acceptor compartments of the perfusion apparatus over the 1 h sampling intervals and containing diclofenac salt, were analyzed using a high-performance liquid chromatograph (Dionex P580 Pump HPLC, Middleton, WI, USA). Aliquots of 20 μL from each sample were injected directly into the column (Phenomenex, C18 (300 mm × 4 mm, 3 μm)). Elution was carried out at room temperature with a mobile phase consisting of a mixture (55:45, *v*/*v*), containing methanol and water, adjusted to pH 2.2 with phosphoric acid; the flow rate was 0.6 mL/min. Detection was performed at 276 nm, using a variable-wavelength UV detector. 

### 3.8. *In vitro* Data Treatment

A calibration curve (peak area *versus* drug concentration) was constructed by running standard drug solutions in PBS for each salt. In the *in vitro* testing, as a result of the sampling of very small volumes (20 μL) from the receiver solution, the replacement of these amounts with equal volumes of buffer was not necessary and the receiver solution volume (100 mL) could be considered constant. Permeation profiles were constructed plotting the cumulative mass of diffusant, m, passing per unit area across the membrane, *versus* time: after a certain period of time (lag time) the graph approaches linearity. To consider factors affecting drug permeation rate through the stratum corneum, that is the transport of the drug across the skin via steady state passive diffusion, the following permeation parameters were evaluated and discussed: lag time (*h*), flux (*J*, μg cm^−2^ h^−1^) and permeability coefficient (*D*, cm/h). The lag time was determined as the origin intercept, extrapolated from the linear portion of the permeation profile. The flux *J* was determined as the slope of the linear portion of the plot. Permeability coefficient was calculated as the ratio of the flux *J* to the concentration of the releasing phase—that is, the solubility value of each salt, the releasing phase being a saturated solution. It was assumed that the drug concentration in the receiver compartment is negligible compared to that in the donor compartment. The values thus obtained for each diclofenac salt are reported in Table 1.

## 4. Conclusions

• Diclofenac permeates porcine membrane when in the form of salts with simple aliphatic amines and interesting fluxes could be measured when the salts are released from saturated aqueous solution;
• Permeation coefficient was found higher when the counterion contains a ring; while hydroxy groups alone do not appear to play an important role, the ring could sustain permeation, disrupting the organized domains of the membrane;
• Different chemical species (acid, anion, ion-pair) contribute to permeation of the anti-inflammatory agent, even though ion-pairs could be hypothesized to operate to a greater extent;
• Potential toxicity of the aliphatic amines, since their cation accompanies the active moiety during the absorption of the ion pairs, cannot be ignored in evaluating the permeation results.
